# Accelerated simulation of unfolding and refolding of a large single chain globular protein

**DOI:** 10.1098/rsob.120087

**Published:** 2012-07

**Authors:** Gavin M. Seddon, Robert P. Bywater

**Affiliations:** 1 Adelard Institute, Manchester M29 7FZ, UK; 2 Magdalen College, Oxford OX1 4AU, UK

**Keywords:** protein structure, protein folding, enzyme reactivation, molecular dynamics

## Abstract

We have developed novel strategies for contracting simulation times in protein dynamics that enable us to study a complex protein with molecular weight in excess of 34 kDa. Starting from a crystal structure, we produce unfolded and then refolded states for the protein. We then compare these quantitatively using both established and new metrics for protein structure and quality checking. These include use of the programs Concoord and Darvols. Simulation of protein-folded structure well beyond the molten globule state and then recovery back to the folded state is itself new, and our results throw new light on the protein-folding process. We accomplish this using a novel cooling protocol developed for this work.

## 2. Introduction

Globular proteins are known to be only marginally stable [1] and some can even operate naturally in a semi-folded state [2]. Thus, any attempt to comprehensively explore the free-energy surface for globular proteins ought to include a study of both the unfolding and refolding process. Several successful attempts to model these processes *in silico* using molecular dynamics (MD) have been published [3–6]. Usually, these have required the use of protracted computer simulations that require abundant computing resources. We report a work that considerably contracts the amount of compute time needed.

In a classical piece of work on the denaturation and refolding of the enzyme ribonuclease [7], the authors showed that full activity could be recovered in the absence of any components of the cellular machinery. This gave rise to the well-known Anfinsen postulate that all information required to attain the correct secondary and tertiary structure is contained in the sequence itself. We have conducted MD simulation studies in order to explore, using simulation, how globular proteins behave when subjected to denaturation, and then renaturation, conditions. While this kind of work has been carried out many times both *in vitro* and *in silico*, we took upon ourselves a major challenge, namely to study an enzyme that is much larger than has heretofore been studied by MD methods.

Our interest is in studying protein unfolding and refolding, not fold prediction as such. Whatever is the particular focus of the work, methods to rapidly traverse the energy landscape of proteins are important. Studies of protein folding by MD simulation were long considered beyond reach, technically, because of limitations in processor speed and memory. A radical breakthrough was achieved by Duan & Kollman [3] who performed a simulation of protein folding for the duration of 1 μs. More recent examples of long MD simulations include work on folding and unfolding on timescales from nanoseconds to microseconds [4], tens of microseconds [5] and even up to milliseconds [6], the latter constituting an impressive world record. It should be pointed out, however, that all of this work was performed either on very small proteins with molecular weight (MW) approximately 4 and 6.5 [6], 7.6 [3] and 7.5 kDa [4], respectively, with one example of a somewhat larger protein with MW 18.6 kDa [5]. These published studies have the merit of providing valuable insights into the folding process, and it is particularly encouraging to note that simulations have in some cases been validated experimentally [4,6]. These studies have not only followed the unfolding and refolding process, but in some instances have also enabled the identification of transition states along these pathways. We note however that some authors claim to be able to return to the starting crystal structure, but we express doubt over whether that is desirable, or even possible, given that the crystal structure contains defects due to crystal packing forces [8] that are very hard to recreate by calculation.

We have developed tools that enable us to design conditions that allow the simulation to proceed more efficiently, in particular a refolding strategy that employs a novel cooling protocol. We successfully conduct and complete a trajectory starting from a crystal structure, through to the denatured state and back to a fully folded state in less than 8 ns, rather than many microseconds, and this with a considerably larger and more complex protein than previously studied elsewhere. We accomplish this using a widely available MD program that has not been fine-tuned to be compatible with any particular computer architecture, Amber 11. The Amber program was initially produced and developed by Case *et al*. [9].

We analyse our results using both well-established protein quality criteria, including packing density, backbone, torsion and bond quality checks and handedness as well as an innovative structure generation program, Concoord. All these are implemented in the What if program as described in §5. In addition, a program, Darvols, was used to track developments in distances between the alpha carbon (CA) atoms of the *i*th and *i*th + 2 residues, areas of triangles subtended by the CA atoms of the *i*th, *i*th + 2 and *i*th + 3 residues, and volumes generated by CA atoms of the *i*th, *i*th + 2, *i*th + 3 and *i*th + 4 residues.

## 3. Results

**Figure 1.**
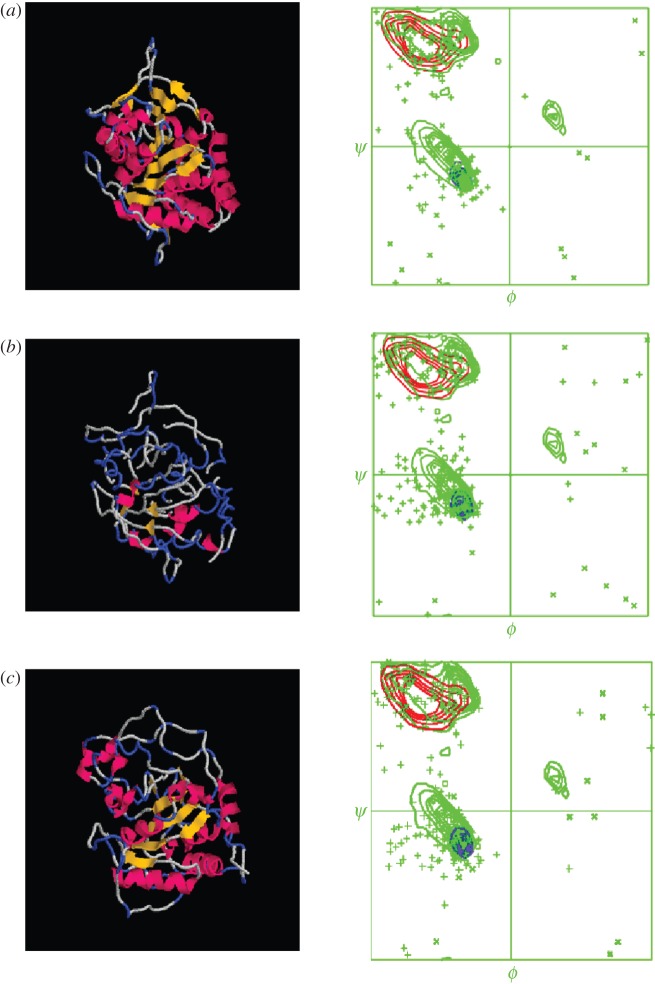
A cartoon showing how the crystal structure first unfolds and then later is refolded, recovering almost all of its original secondary structure. The Ramachandran diagrams accompanying each cartoon image display the folding information in more detail.

**Figure 2.**
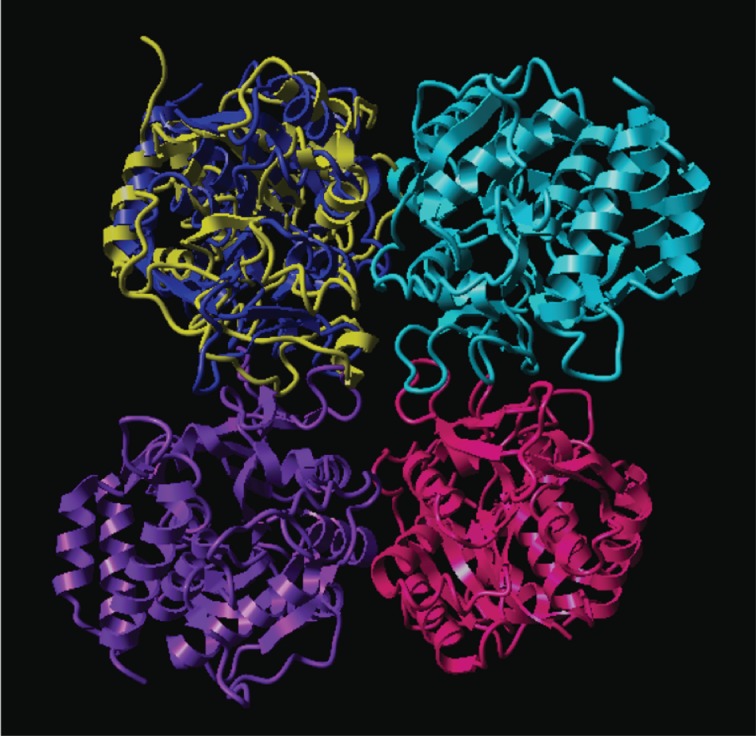
The refolded structure is superimposed onto one of the monomers of a crystallographic tetramer to reveal overlaps with neighbouring monomers (see §4).

**Figure 3.**
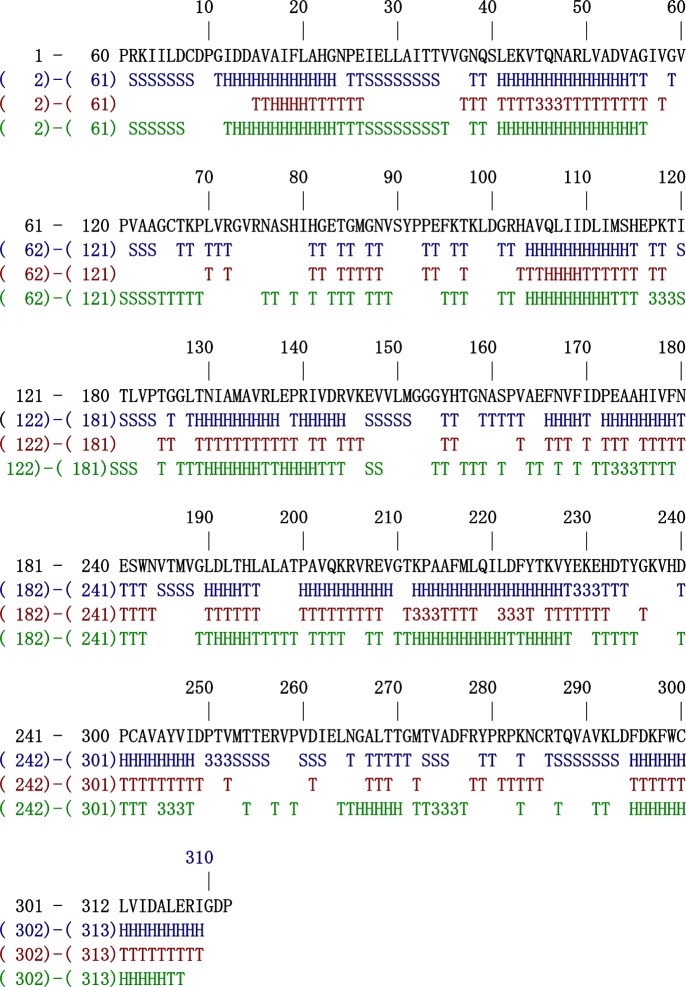
All secondary structure elements that were originally present in the crystal structure, which became completely eradicated in the unfolding step, have been recovered.

**Figure 4.**
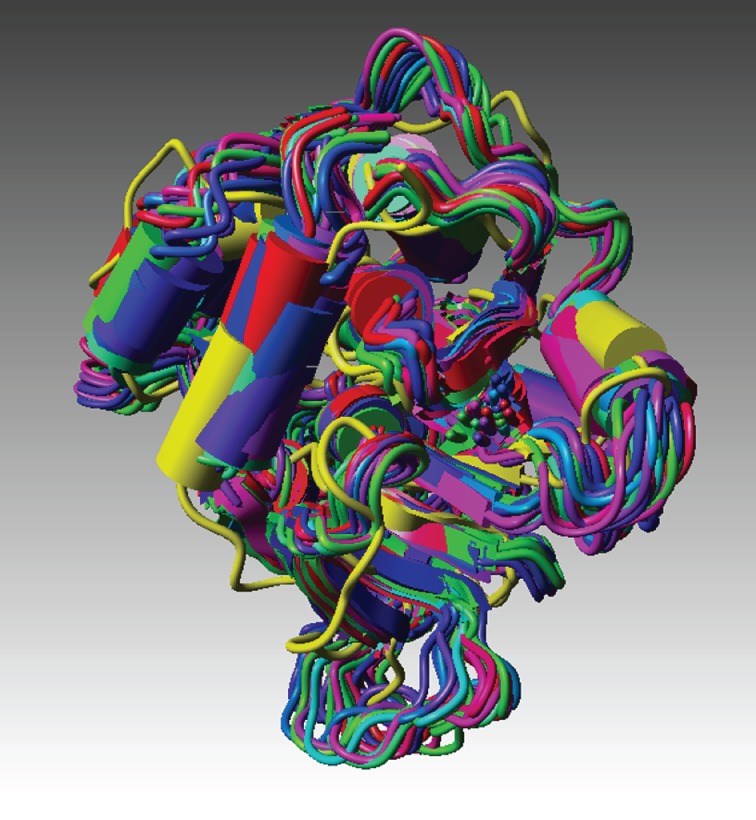
The final refolded structure embedded in an ensemble of structures derived from the original crystal structure using the Concoord procedure (see §4).

We report MD simulations on nucleoside N-ribohydrolase starting from its crystal structure, under thermal denaturation conditions to a state where secondary structure has been almost entirely obliterated. We then alter the conditions to allow the protein to refold, and find that the protein recovers almost all its initial secondary structure, while its core packing and other quality control parameters show a protein structure that is clearly in good health. We accomplish refolding of a large protein within less than 8 ns and do not see the need to strive for ever longer simulation times. The results are summarized in figures 1–4 and tables 1 and 2.

**Table 1.** Key structural parameters and quality checks of the starting structure, the denatured structure and its refolded counterpart.

structure	total cavity volume (Å^3^)^a^	average area (Å^2^)^b^	average volume (Å^3^)^b^	% helix^c^	% 3_10^c^	% strand^c^	% turn^c^	% coil^c^	BBCCHK^d^	QUACHK^d^	RAMCHK^d^	HNDCHK^d^	r.m.s. comparison^e^
crystal	148.36	1716.92	2521.74	37.18	1.92	15.71	19.23	25.96	0.14	−0.75	−2.42	0.59	0
ezrU1000	135.94	1875.24	3020.59	25.00	2.24	10.90	33.01	28.85	−3.14	−1.91	−6.59	4.81	1.56
ezrU2000	102.06	1846.57	2837.62	18.59	4.81	8.97	36.22	31.41	−3.95	−2.08	−7.72	6.52	1.92
ezrU3000	143.39	1870.54	2862.15	5.77	2.89	3.21	49.36	38.78	−3.51	−2.33	−7.97	7.88	2.51
ezrU4000	148.41	1926.01	3027.20	7.69	1.92	4.81	46.47	39.11	−4.48	−2.69	−8.39	8.78	2.77
ezrU5000	228.08	1856.60	2855.86	2.56	2.89	0	48.40	46.15	−3.90	−2.73	−8.91	10.11	2.98

^a^Cavity volume (Cavvol) analyses for the protein in starting, unfolded and refolded state.

^b^darvols areas and volumes, respectively.

^c^The secondary structure statistics.

^d^Backbone conformation normality check (BBCCHK), packing quality control (QUACHK), Ramachandran *z*-score (RAMCHK) and checks for atoms with the wrong hand (HNDCHK), respectively.

^e^Results of r.m.s. comparison of simulation structures compared with the starting crystal structure.

**Table 2.** Key structural parameters and quality checks of the starting structure, a single denatured structure and refolded structures.

structure	total cavity volume (Å^3^)^a^	average area (Å^2^)^b^	average volume (Å^3^)^b^	% helix^c^	% 3_10^c^	% strand^c^	% turn^c^	% coil^c^	BBCCHK^d^	QUACHK^d^	RAMCHK^d^	HNDCHK^d^	r.m.s. comparison^e^
crystal	148.36	1716.92	2521.74	37.18	1.92	15.71	19.23	25.96	0.14	−0.75	−2.42	0.59	0
ezrU5000	228.08	1856.60	2855.86	2.56	2.89	0	48.40	46.15	−3.90	−2.73	−8.91	10.11	2.98
5 ns refold	171.01	2061.87	3288.21	26.28	0.96	5.13	33.65	33.97	−2.63	−2.15	−5.41	2.45	6.18
7.833 ns	136.29	1845.42	3061.50	25.96	3.85	6.73	31.73	31.73	−2.56	−2.06	−5.17	2.56	5.95

^a^Cavity volume (Cavvol) analyses for the protein in starting, unfolded and refolded state.

^b^darvols areas and volumes, respectively.

^c^The secondary structure statistics.

^d^Backbone conformation normality check (BBCCHK), packing quality control (QUACHK), Ramachandran *z*-score (RAMCHK) and checks for atoms with the wrong hand (HNDCHK), respectively.

^e^Results of r.m.s. comparison of simulation structures compared with the starting crystal structure.

Figure 1, which was produced using the protein graphics program Rasmol [10,11], shows in cartoon form how the crystal structure first unfolds and then later is refolded, recovering almost all of its original secondary structure (see also figure 3 and table 1). The Ramachandran diagrams accompanying each Rasmol image display the folding information in more detail. The loss, and later recovery, of secondary structure is manifest in these diagrams, as is the appearance of points representing residues in torsional states that are well outside the normal envelope(s) for secondary structures and turns. Note that we do not expect the final structure to be exactly like the crystal structure, because crystal structures contain artefacts that arise from packing constraints in the crystal lattice, as has been noted elsewhere [8]. In figure 2, the refolded structure is superimposed onto one of the monomers of a crystallographic tetramer in order to reveal that overlaps with neighbouring monomers can be discerned (see §4).

Figure 3 shows a most significant item of data which demonstrates that we have really embarked on a refolding pathway and arrived at a properly folded structure. Each and every one of the secondary structure elements that were originally present in the crystal structure that became completely eradicated in the unfolding step has been recovered. This is a tribute to the force field we use, which faithfully reproduces the actual potential field felt by the atoms in the protein as well as the desolvation events necessary to drive the folding process, but also to our management of the temperature throughout the recovery process. It is our contention that no researchers have heretofore succeeded in simulating unfolding of a protein to the extent that secondary structure is to all intents and purposes entirely abrogated, only to have this same secondary structure restored.

Figure 4 shows our final, refolded structure embedded in an ensemble of structures derived from the original crystal structure using the Concoord procedure [12] implemented in What if [13]. This procedure identifies conformations that are energetically accessible from a given starting structure.

## 4. Discussion

As can be seen from the results in tables 1 and 2, the protein undergoes considerable deformations to begin with, before finally being restored to a compact, well-formed, three-dimensional structure. In particular, it is noticeable that the final structure is more compact than the starting crystal structure according to the Cavvol metric. The Darvols results provide another measure of compactness, more to do with how packing is distributed along the polypeptide chain. It is noticeable that these two metrics do not return to the values for the original crystal structure but neither does the secondary structure content reproduce the original values. The various quality checks show a decline in quality as the protein unfolds and steadily loses secondary structure, which then recovers as the secondary structure recovers. It should be stressed that these quality checks are based upon crystal structure data (not water-soluble proteins) that can be much more flexible. The r.m.s. data for the refolded structures show that the structure returns to something close to the crystal structure that it started from, but of course we do not expect it ever to return to that structure for reasons stated earlier in this work.

The Ramachandran diagrams for our various structures are shown in figure 1 alongside cartoons of the structures. These Ramachandran structures show how well-formed the final structure is; in particular, there are no more residues in disfavoured states than there are in the starting crystal structure.

Figure 2 illustrates the point already made, that, given the freedom to move in solution, the protein encroaches on space which, in the crystal, would be occupied by its neighbours. The regions that make this excursion are also mirrored in the Darvols area and volume plots (not shown, but can be obtained as three-dimensional rotatable Gnuplot images from the authors).

Figure 3 demonstrates that each and every one of the secondary structure elements in this large, complex protein has been reconstructed. There is no precedent for this in previously published work. But we see that every secondary structure element has been found. The full extent of rewinding in some instances may have not been fully recovered (table 2) but neither do we expect this to happen when the protein has to exist, or rather, coexist, with many other members of the ensemble of accessible structures.

Figure 4 is perhaps the most telling of all because it shows our final, refolded structure embedded in an ensemble of 25 structures derived from the original crystal structure. These 25 structures are the ones most immediately accessible to the crystal structure, once it is free from the confines of its crystal environment. Our final (refolded) structure embeds comfortably within the envelope formed by these structures, apart, that is, from some of the extremities of some of the loops. But then we have already explained why some flexible regions of the protein in solution will not conform to the more constrained crystal structure. What is significant about this figure is that it shows the close relationship, conformationally, between our final structure and this envelope of possible structures. Although there are considerable distortions in crystal structures owing to lattice packing constraints, it is significant that our structures are not all that remote from the crystal structure. The quantitative ‘remoteness’ can be judged from the r.m.s. displacement data presented in table 3. This shows that the structure does, of course, not revert to the original crystal structure. Neither would we expect it to. But it is sufficiently close, as shown in our set of superimposed structures. There is also the issue of which structure it should revert to. This is discussed later.

**Table 3.** Root mean square displacements between structures calculated by the Concoord program and between the final folded structure and the Concoord structures. ‘input’ denotes original crystal structure for all simulations, 1ezr.pdb; ‘consensus’ denotes consensus or ‘best’ representative structure in the Concoord set.

Concoord structure	r.m.s. displacements between Concoord structure and input structure	r.m.s. displacements between Concoord structure and final structure
1	2.23171	5.7630
2	2.07303	5.3450
3	1.34048	5.5060
4	1.41400	5.1950
5	1.71137	5.5820
6	1.43210	5.4920
7	1.14649	5.2010
8	2.04711	5.6530
9	1.87252	5.2870
10	1.58538	4.9960
11	2.04818	5.3510
12	1.71161	5.1960
13	1.57967	5.5000
14	1.23916	5.2460
15	1.60457	5.3560
16	1.49080	5.2460
17	1.55956	5.3470
18	1.58217	5.4190
19	1.68927	5.6910
20	1.82766	4.9040
21	1.76882	5.4940
22	1.65827	5.4550
23	1.49889	5.3740
24	1.82959	5.5420
25	2.16817	5.8710
	input	5.1900
	consensus	5.3740

In the present work we study protein refolding, defined herein as the process whereby a folded protein in its equilibrium state undergoes denaturation to a point where the unfolding can still be reversed, which we also demonstrate. This is of course only part of the total protein-folding agenda. The entire trajectory from nascent polypeptide to final structure, the principal focus of *ab initio* fold prediction, obviously requires the study of more steps than we have accomplished here. We have also embarked on this latter work, which will be reported separately.

The folding simulations by other authors referred to earlier dealt with refolding from an unfolded state and it was stated [3] that they achieved a ‘state similar to the native state’ after only 1 ms. Elsewhere [6], the ‘native state’ was claimed to have been reattained. It was not made clear in either case how similar their refolded state was to any starting structure (crystal structure or alternative), and anyway we prefer to eschew references to ‘the’ native state, preferring the notion of equilibrium folded state. The concept of ‘the’ native structure needs to be reconsidered in the light of data that shows that proteins can adopt one of several energetically accessible structures [14]. (Anyway, ‘native’ implies ‘having just been born’, but we are concerned here with the final stage of the folding process, not the protein synthesized de novo on the ribosome.) Our unfolded state is derived from a crystalline state that we denature and then recover to a stable equilibrium structure in water with a well-packed (column 2 in table 2) three-dimensional structure. Our structure is now a minimum energy structure devoid of all traces of crystal packing artefacts. Thus, we show here, for the first time, that it is possible to simulate the unfolding of a protein and then refold it. Our methodology can easily be reproduced and run routinely with bigger proteins. The calculations are estimated to scale as *O*(*n^k^*) with 1 < *k* < 2 depending on the MD algorithm used, and the way in which the program is mapped to the hardware. Further, we use a widely available force field (Amber 11) that can be used by anybody.

We have explored only part of the free-energy landscape that the protein has to negotiate on its way from biosynthesis to its final natural state. An important intermediate on the folding pathways is the molten globule [15], and we are cognizant of the fact that not all proteins fold into a compact form [2], whereas others (the majority) can coexist in at least two conformations [14,15] depending on conditions such as the presence or absence of ligands. Recent studies [16] have highlighted the sometimes very considerable flexibility in proteins when performing their assigned cellular functions. In the light of these studies, we do not expect our protein to return to the crystal structure, but the notion that our structure could represent one of the structures to be found in the ensemble of active conformations is very appealing and gives us occasion to perform further studies. In the context of structural biology, our final structure may resemble the kind of structure obtained by NMR. We have embarked on a study of exactly this question and will report the results shortly in a companion study.

We think it is important to study proteins where there are no disulphide bridges as well as disulphide-bond-stabilized proteins. As Anfinsen [7] himself stated ‘After several years of study on the ribonuclease molecule, it became clear to us and to many others in the field of protein conformation that proteins devoid of restrictive disulfide bonds or other covalent cross-linkages would make more convenient models for the study of the thermodynamic and kinetic aspects of the nucleation, and subsequent pathways, of polypeptide chain folding’. This is the logic behind the present study, but we are also reproducing the Anfinsen experiment itself, the refolding of ribonuclease, but now conducted *in silico*, which is reported separately [17].

We contend that our approach provides useful insights into the folding process, and it can be used to design experiments. One advantage that mathematical and computational methods will always have over experimental methods is that they are non-intrusive, unlike many experimental techniques, for example laser irradiation, with a concomitant risk for radical formation and photolytic cross-linking, and, in crystallography, the crystal packing defects [8] already alluded to. In particular, we note that there is now no great computational barrier to studying large proteins such as the one we have reported on here. Very likely, the continued development of hardware according to Moore's Law [18] will make this technology ever more widely available to protein chemists. We show, however, that one does not need to wait for this development to take place. As long as one is interested only in the endpoints of the trajectory and not the trajectory itself, our approach offers a new strategy as to how to steer the refolding process from unproductive dead-ends back onto a successful refolding pathway. We do not claim that our approach can routinely be used to accomplish *ab initio* folding. Our results support the notion that sequence determines fold, but, while our unfolding removes almost all traces of secondary structure, there may well be sufficient ‘memory’ left in the structure to ensure that the structure can recover a full complement of secondary structure.

## 5. Methods

### 5.1. Selection of protein to study

The nucleoside N-ribohydrolase from *Leishmania major* (EC 3.2.2.1) was chosen because it is a protein that is both large (312 residues, MW 34 kDa) and complex (alpha + beta fold). It is a single-chain protein, chosen so that only intramolecular interactions need be considered. The starting structure was the X-ray crystal structure (PDB id 1ezr).

### 5.2. Molecular dynamics simulations

All simulations were conducted using the Amber 11 force field [9] as implemented in the protein-modelling package Yasara [13], in tip4p water, solvent density 0.997 g ml^−1^, at constant pressure 1013 hPa.

Initially, unfolding (thermal denaturation) was achieved by heating to 5000°C for 1.0 ps, by which time all secondary structure elements were completely lost (figure 1*a*).

Refolding was induced at elevated temperature, 300°C, followed by a cooling protocol for 1.2 ns, 200°C for 3.7 ns and 100°C for 0.1 ns. Simulations were saved periodically every 0.05 ns. The rationale behind the design of this protocol was that a critical temperature needed to be reached that corresponds to a level inside the free-energy surface (‘funnel’) that is sufficiently high to allow the protein to descend into its correct fold during cooling. At elevated temperature, torsional rotation is increased and hence the protein samples configurations to find the lowest, most stable primary sequence configuration and relaxes to a more stable secondary and tertiary geometry. Varying temperatures were used here in order rapidly to reach a more stable conformation. We noted that an increase of approximately 100°C halved the search time within the temperature landscape or range chosen. We found that the consequences were identical whatever thermal parameters were used. The same refolding procedure was performed under different temperature settings. The refolding followed the same pathway, only the speed was different. At higher temperatures, folding is faster but instability is reached sooner.

The entire trajectory was concatenated to create a movie (http://www.adelard.org.uk/movies).

### 5.3. Determination of some key metrics of the protein folding

These were all determined using the protein-modelling package What if in the twinned version together with the Yasara program [13]. The secondary structure composition (as per metrics cent α-helix, 3_10_ helix, strand, turn and coil) was determined using the What if program. Quality checks used here were backbone conformation normality check, packing quality control, Ramachandran Z-score and chirality checks.

A noticeable feature of correct three-dimensional folding of globular proteins is that they are very compact, whereby voids and internal waters occur only rarely. We checked the compactness of the structures obtained by calculating the volumes of both the largest cavity, and the sum of volumes of all cavities in the protein using What if.

### 5.4. Computer programs to analyse the trajectories

In order to detect memories of previous folding arrangements, computer programs were written that determine how packing is distributed along the length of the polypeptide chain. These programs are written in Fortran and they compile under gFortran or g77. They are archived on our website and can be downloaded from there.^1^

The program Darvols performs a number of geometrical calculations on the protein of which some are useful for determining how the packing is distributed along the polypeptide chain (of *N* residues). These packing metrics include the areas of triangles formed by CA atoms spaced two residues apart in succession and the global centre of gravity (CoG) of the protein and the volumes of tetrahedra formed by three CA atoms spaced two residues apart in succession and the global CoG. The results of these calculations appear in tables 1 and 2.

### 5.5. Procedure for finding rapid refolding conditions

The refolding was accomplished in several stages, because we saw the need to alter conditions at certain points along the recovery trajectory. The recovery was started at an elevated temperature, 300°C, until instability of the protein was noted visually and also as judged by tracking the development of areas and volumes as determined by the Darvols program. Increases in these parameters reflect deterioration of protein packing. Accordingly, the MD temperature was reduced to 200°C until a similar event occurred. Again, the temperature was reduced, this time to 100°C until a total time of 7.833 ns. At this point, no further improvements could be made, but we had regained structural integrity with good packing and all the secondary structure elements present in the initial structure were recovered, albeit not fully (table 1).

## 7. Endnote

1 Movies and MD trajectories related to this study can be downloaded from www.adelard.org.uk/movies. The Darvols program can be supplied upon application by email to author R.P.B.

## References

[1] Fersht A 1999 Structure and mechanism in protein science, pp. 508–509 New York, NY: WH Freeman & Co

[2] Uversky VN 2002 Natively unfolded proteins: a point where biology waits for physics. Protein Sci. 11, 739–756 10.1110/ps.4210102 (doi:10.1110/ps.4210102) 11910019 PMC2373528

[3] Duan Y Kollman PA 1998 Pathways to a protein folding intermediate observed in a 1-microsecond simulation in aqueous solution. Science 282, 740–744 10.1126/science.282.5389.740 (doi:10.1126/science.282.5389.740) 9784131

[4] Mayor U 2003 The complete folding pathway of a protein from nanoseconds to microseconds. Nature 421, 863–867 10.1038/nature01428 (doi:10.1038/nature01428) 12594518

[5] Freddolino PL Liu F Gruebele M Schulten K 2008 Ten-microsecond molecular dynamics simulation of a fast-folding WW domain. Biophys. J. 94, L75–L77 10.1529/biophysj.108.131565 (doi:10.1529/biophysj.108.131565) 18339748 PMC2367204

[6] Shaw DE 2010 Atomic-level characterization of the structural dynamics of proteins. Science 330, 341–346 10.1126/science.1187409 (doi:10.1126/science.1187409) 20947758

[7] Anfinsen CB 1973 Principles that govern the folding of protein chains. Science 181, 223–230 10.1126/science.181.4096.223 (doi:10.1126/science.181.4096.223) 4124164

[8] Peters G Bywater RP 2002 Essential motions in a fungal lipase with bound substrate, covalently attached inhibitor and product. J. Mol. Recognit. 15, 393–404 10.1002/jmr.579 (doi:10.1002/jmr.579) 12501159

[9] Case DA 2010 AMBER 11. San Francisco, CA: University of California

[10] Sayle R Milner-White EJ 1995 RasMol: biomolecular graphics for all. Trends Biochem. Sci. 20, 374 10.1016/S0968-0004(00)89080-5 (doi:10.1016/S0968-0004(00)89080-5) 7482707

[11] Bernstein HJ 2000 Recent changes to RasMol, recombining the variants. Trends Biochem. Sci. 25, 453–455 10.1016/S0968-0004(00)01606-6 (doi:10.1016/S0968-0004(00)01606-6) 10973060

[12] De Groot BL Van Aalten DMF Scheek RM Amadei A Vriend G Berendsen HJC 1997 Prediction of protein conformational freedom from distance constraints. Proteins 29, 240–251 10.1002/(SICI)1097-0134(199710)29:2<240::AID-PROT11>3.0.CO;2-O (doi:10.1002/(SICI)1097-0134(199710)29:2<240::AID-PROT11>3.0.CO;2-O) 9329088

[13] Krieger E Koraimann G Vriend G 2002 Increasing the precision of comparative models with YASARA NOVA: a self-parameterizing force field. Proteins 47, 393–402 10.1002/prot.10104 (doi:10.1002/prot.10104) 11948792

[14] Bywater RP In press. Protein folding: a problem with multiple solutions. J. Biomol. Struct. Dyn. 10.1080/07391102.2012.703062 22870987

[15] Ptitsyn OB 1995 Molten globule and protein folding. Adv. Protein Chem. 47, 83–229 10.1016/S0065-3233(08)60546-X (doi:10.1016/S0065-3233(08)60546-X) 8561052

[16] Yang S Blachowicz L Makowski L Roux B 2010 Multidomain assembled states of Hck tyrosine kinase in solution. Proc. Natl Acad. Sci. USA 107, 15 757–15 762 10.1073/pnas.1004569107 (doi:10.1073/pnas.1004569107) PMC2936629 20798061

[17] Seddon GM Bywater RP 2012 Inactivation and reactivation of ribonuclease A studied by computer simulation. Open Biol. 2, 120088 10.1098/rsob.120088 (doi:10.1098/rsob.120088) 22868279 PMC3411109

[18] Moore GE 1965 Cramming more components onto integrated circuits. Electronics Mag. 8, 4–7

